# Sorption Hysteresis of Light Hydrocarbons and Carbon Dioxide in Shale and Kerogen

**DOI:** 10.1038/s41598-017-13123-7

**Published:** 2017-11-24

**Authors:** Huangjing Zhao, Zhiping Lai, Abbas Firoozabadi

**Affiliations:** 1 Reservoir Engineering Research Institute, 595 Lytton Avenue Suite B, Palo Alto, CA 94301 USA; 2 Advanced Membranes & Porous Materials Center, King Abdullah University of Science and Technology, Thuwal, 23955-6900 Saudi Arabia

## Abstract

We present adsorption and desorption isotherms of methane, ethane, propane, *n*-butane and *iso*-butane as well as carbon dioxide for two shales and isolated kerogens determined by a gravimetric method. The sorption measurements of two shales were performed at three different temperatures, 308.15, 323.15, and 338.15 K. For the isolated kerogens, the measurements were conducted at 338.15 K. Methane and ethane sorption isotherms were measured to 35 bar. Carbon dioxide sorption isotherms were studied to 30 bar. Due to the low vapor pressure at room temperature, the sorption isotherms of propane, *n*-butane and *iso*-butane were measured to 8, 2, and 2 bar, respectively. The adsorptions of propane, *n*-butane, and *iso*-butane were much higher than methane at the highest pressures where the measurements were conducted. The adsorption of *n*-butane was 10 times higher than methane by mole at 2 bar, followed by *iso*-butane and propane. Our data show significant adsorption hysteresis in ethane, propane, *n*-butane and *iso*-butane. The most pronounced hysteresis was found in *n*-butane and *iso*-butane. Significant hysteresis is attributed to the reversible structural changes of kerogens. Dissolution of adsorbates into organic matter may also affect the hysteresis. This is the first report of propane and butane sorption isotherms in shales.

## Introduction

Natural gas, the cleanest fossil fuel and the premium fuel of the twenty-first century, is desirable for various uses. Electrical power generation by natural gas is highly desirable environmentally. Use of natural gas in transportation systems can reduce carbon emissions. Current limitation is storage of natural gas in heavy cylinders in vehicles. New materials such as flexible metal-organic frameworks with higher adsorption capacity may enhance the use of natural gas in vehicles drastically. Around 30% of the world’s natural gas resource is from shales. Shale gas may profoundly affect alternative energies because of the potential wide use^1^.

Shale gas and light-oil formations are fundamentally different from conventional and tight gas formations. Conventional and tight gas formations are composed of permeable media with pore sizes greater than 100 nm. Shale is composed of two distinct media, organic and inorganic. The important feature of shale is the nanoscale size of pores, in both organic and inorganic matters. The organic materials are made of molecules with a large number of aromatic rings with heteroatoms and alkane tails. They can be divided into extractable organic components (such as bitumen) by organic solvents, and insoluble macromolecular organic matter (such as kerogen). Kerogen is the predominant part of organic matter in most shales^2^. Generally, with higher content of organic matter, the sorption of hydrocarbon is higher. The inorganic matter of shale has similarities to conventional formations and contains clay, calcite, quartz, etc.

Adsorption of fluid species on the inner surface of pores may contribute significantly to total fluid-in-place when the ratio of surface area to the pore volume increases^3,4^. Due to the nanoscale pores in shale, adsorption can be an important part of total species-in-place. Fluid-in-place may be divided by three categories: free molecules in the pores; adsorbed species on the inner surfaces of the microscale and nanoscale pores; and dissolved species in the organic matter^5^.

There are two common methods to measure gas adsorption, gravimetric and volumetric/manometric^5–14^. Gravimetric methods use high-resolution balances to directly measure the weight change of samples due to sorption. The volumetric/manometric methods are based on Boyle’s law. In this investigation, the Rubotherm Sorption System was used to measure sorption isotherms of various light hydrocarbons and carbon dioxide in shales and isolated kerogens. The Rubotherm Sorption System contains a magnetic suspension balance to accurately measure the weight change of sample materials affected by gravity and buoyancy in the sorption measurements. In gravimetric methods, one can accurately measure the uptake of gas (which does not include free gas in the pores). Adsorption and dissolution cannot be differentiated. Two different shale samples were selected in this study. The outcrop shale sample contains over 50 wt% total organic matter, and the reservoir shale has around 3.6 wt% total organic matter which is typical in many shale formations.

The maximum pressure of the set-up is 40 bar. We investigated the sorption isotherms of methane, ethane and carbon dioxide in the two shale samples to 35, 35, and 30 bar, respectively, at three different temperatures, 308.15, 323.15, and 338.15 K. Unlike methane and carbon dioxide, there is only one published report on ethane adsorption in shales. Recently, Gasparik *et al*. have reported ethane adsorption in the Upper Chokier and Lower Toarcian shales^6^. Due to the low vapor pressures of propane, *n*-butane and *iso*-butane at room temperature, we performed our measurements of sorption isotherms of propane, *n*-butane and *iso*-butane to 8, 2, and 2 bar, respectively. To the best of our knowledge, there are no reported adsorption measurements of propane and butanes in shales. The sorption measurements of isolated kerogens were conducted at 338.15 K.

There is limited work on desorption in shale media for methane and carbon dioxide, despite the fact that desorption is more relevant in production of shale gas. Yuan *et al*. have reported methane adsorption/desorption isotherms on dry and moist shale sample from Sichuan Basin, China^13^. They observed a slight hysteresis in dry shale and a strong hysteresis in moist shale. Hysteresis in gas adsorption/desorption is generally attributed to capillary condensation in mesoporous materials^4^. If capillary condensation is applicable, there should be no hysteresis in methane and carbon dioxide adsorption at the temperatures of this study. However, in coal seams a number of laboratory studies have reported hysteresis in methane and carbon dioxide adsorption^15–28^. The mechanism of hysteresis of methane and carbon dioxide in adsorption in coal is an open question, and many possible links have been suggested including moisture in the coal sample, surface geometry heterogeneity, chemical interaction, structural deformation, and insufficient equilibration time^29^. In heavier hydrocarbons, the mechanism of sorption hysteresis can be linked to the critical temperature^4^. We have measured sorption isotherms of various hydrocarbons and carbon dioxide in dry conditions for shale media to shed light on hysteresis. Our data reveal significant hysteresis in ethane, propane, *n*-butane and *iso*-butane. Even in methane and carbon dioxide, a measureable hysteresis is observed.

## Results

### Material Characterization

Two different shale samples were investigated in this study. One shale sample (Kimmeridge Blackstone) is from the Blackstone band of the Kimmeridge Clay Formation from an outcrop east of Kimmeridge Bay in Dorset, UK. The other (Neuquén Shale) is from a Neuquén Basin well, Argentina. The mineralogy of the inorganic part of shale samples are dominated by quartz, calcite and clay (Table 1). The thermal maturity in terms of vitrinite reflectance is 0.99% for Kimmeridge Blackstone, which is in the heart of the oil window. The vitrinite reflectance of Neuquén Shale is 1.06%. This value indicates that Neuquén Shale is in the mid- to late-early part of the oil window.

**Table 1:** Basic mineralogy (wt%) of shale samples.

	Quartz	Feldspar	Calcite	Pyrite	Clay	Rest
Kimmeridge Blackstone	10	0	52	7	26	5
Neuquén Shale	19	8	55	1	15	2

**Figure 1:**
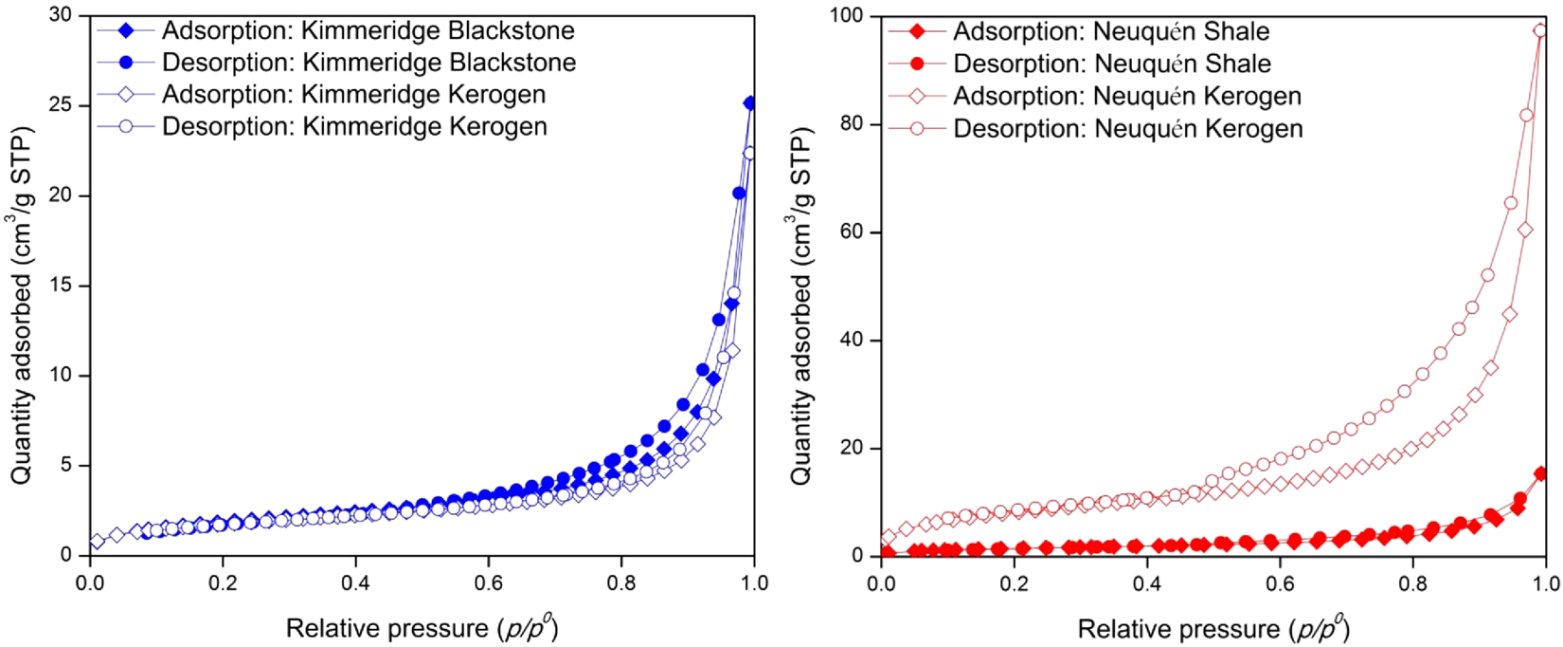
Nitrogen adsorption and desorption in shale and kerogen powder samples at 77.3 K.

Surface area, pore volume, and average pore size of the shale and kerogen powder samples were determined by nitrogen adsorption/desorption at 77.3 K. Surface area was calculated using the Brunauer–Emmett–Teller (BET) model, while pore volume and average pore size were calculated by the Barrett–Joyner–Halenda (BJH) model. Figure 1 shows the nitrogen adsorption/desorption in the shale and kerogen powder samples at 77.3 K.

The BET surface areas of Kimmeridge Blackstone and Neuquén Shale powders are 7.0 m^2^/g and 5.6 m^2^/g, respectively (see Table 2); the BJH pore volumes of Kimmeridge Blackstone and Neuquén Shale powders are 0.038  cm^3^/g and 0.023 cm^3^/g, respectively. The average pore sizes of the isolated kerogen powder samples from Kimmeridge Blackstone and Neuquén Shale are 25.9 nm and 22.8 nm, respectively. The BET surface area and BJH pore volume of the isolated Neuquén Kerogen are significantly higher than Neuquén Shale (5.3 and 6.3 times respectively), because of the removal of the inorganic matter and bitumen (around 95% of total mass) in the kerogen isolation processes. Unlike the isolated kerogen from Neuquén Shale, both BET surface area and BJH pore volume of the isolated Kimmeridge Kerogen are similar to shale. Kimmeridge Blackstone contains more than 50% total organic matter. In the kerogen isolation, apparently the properties of surface and pores of Kimmeridge Blackstone did not change much.

**Table 2:** Surface area, average pore size, and pore volume of shale and kerogen powder samples.

	BET surface area (m^2^/g)	BJH pore volume (cm^3^/g)	BJH average pore size (nm)
Kimmeridge Blackstone	7.0	0.038	25.1
Kimmeridge Kerogen	6.6	0.034	25.9
Neuquén Shale	5.6	0.023	18.8
Neuquén Kerogen	29.7	0.146	22.8

Table 3 shows the CHNS-O elemental analysis. Total carbon (organic and inorganic carbon) and inorganic carbon contents of shale samples were measured, respectively. Kimmeridge Blackstone contains 51.19 wt% total organic carbon, while Neuquén Shale has only 3.65 wt% total organic carbon. Kimmeridge Blackstone contains more than 60 wt% kerogen and Neuquén Shale has around 5 wt% kerogen. These kerogen contents are based on the kerogen isolation process to be discussed in the Methods Section. For the Kimmeridge Blackstone we start with 10 g of shale powder and processing the sample results in about 6.3 g of krogen powder. Kerogen content of the Neuquén shale was based on 10 g of the shale powder and approximate recovery of 0.5 g of kerogen. The isolated kerogens from both Kimmeridge Blackstone and Neuquén Shale have high sulfur content due to the remaining pyrite from the kerogen isolation processes. The Qualitative X-ray Diffraction (XRD) Analysis of the samples provide the evidence. Figure S1 shows the XRD patterns of shale and kerogen powder samples. Samples were scanned from 10 to 80° (2θ). The XRD plots of isolated Kimmeridge and Neuquén kerogen show identical pyrite XRD patterns. If the isolated kerogens were pure, there should be no discernible peak from the powder XRD analysis.

**Table 3:** CHNS-O composition and total organic carbon analysis of shale and kerogen samples.

	H (wt%)	TC^1^ (TOC^2^) (wt%)	N (wt%)	O (wt%)	S (wt%)	Rest (wt%)
Kimmeridge Blackstone	5.45	52.71 (51.19)	1.40	8.84	7.18	24.42
Kimmeridge Kerogen	6.49	62.52 (62.52)	1.70	5.58	11.31	12.40
Neuquén Shale	0.47	8.84 (3.65)	0.14	10.57	0.56	79.42
Neuquén Kerogen	3.63	58.07 (58.07)	1.82	3.01	15.00	18.47

^1^TC: Total Carbon; ^2^TOC: Total Organic Carbon.

From the CHNS-O elemental analysis in Table 3, hydrogen to carbon atom ratio is 1.246 and oxygen to carbon atom ratio is 0.067 in the isolated Kimmeridge Kerogen. Kimmeridge Kerogen may be classified as Type II. In the kerogen isolated from Neuquén Shale, hydrogen to carbon atom ratio is 0.750 and oxygen to carbon atom ratio is 0.039, which indicates that Neuquén Kerogen is Type III^30^. In the organic petrography analysis (Figure S2), however, both samples are interpreted to be of marine origin owing to the presence of abundant fluorescing liptinite maceral in the form of lamalginite, degraded lamalginite and amorphinite. Trace amounts of inertinite maceral were observed in Kimmeridge Blackstone. Small amounts of vitrinite maceral were noticed in Neuquén Shale. Infrared spectroscopy (IR) analysis shows that aliphatic/aromatic ratios in Kimmeridge Blackstone and Neuquén Shale are 9.98 and 1.35, respectively.

The combination of thermal maturity from vitrinite reflectance and kerogen type provides the boundary between oil and gas zones. Based on the information above the shale reservoir is in the oil zone.

### Sorption Measurement

**Figure 2:**
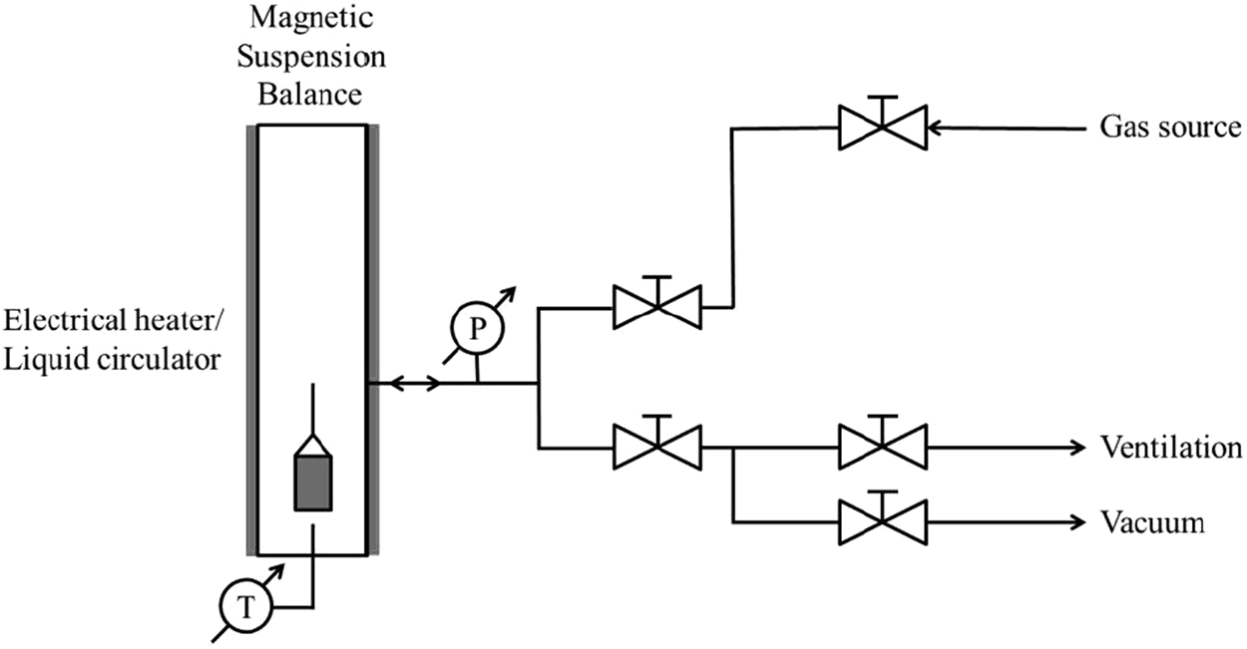
Schematic diagram of the gravimetric gas adsorption analyzer.

Figure 2 shows the schematic diagram of the gravimetric adsorption analyzer. Before the sorption measurements, the densities of Kimmeridge Blackstone, Neuquén Shale and kerogens isolated from shale samples were measured using helium gas at 308.15 K. The densities of Kimmeridge Blackstone and kerogen isolated from Kimmeridge Blackstone are 1.504 g/cm^3^ and 1.345 g/cm^3^, respectively. The densities of the Neuquén Shale and kerogen isolated from Neuquén Shale are 2.607 g/cm^3^ and 1.725 g/cm^3^, respectively. Eseme *et al*.^31^ report that density of pure kerogen is in the range of 1.0 g/cm^3^ to 1.3 g/cm^3^. However, both of the isolated kerogens from Kimmeridge Blackstone and Neuquén Shale have higher densities; they may contain pyrite which has a density of 4.8–5 g/cm^3^. This is confirmed by the powder XRD analysis shown in Figure S1. Heavy minerals present in the isolated kerogens, such as rutile, tourmaline, and zircon, may also contribute to the higher densities^32^.

**Figure 3:**
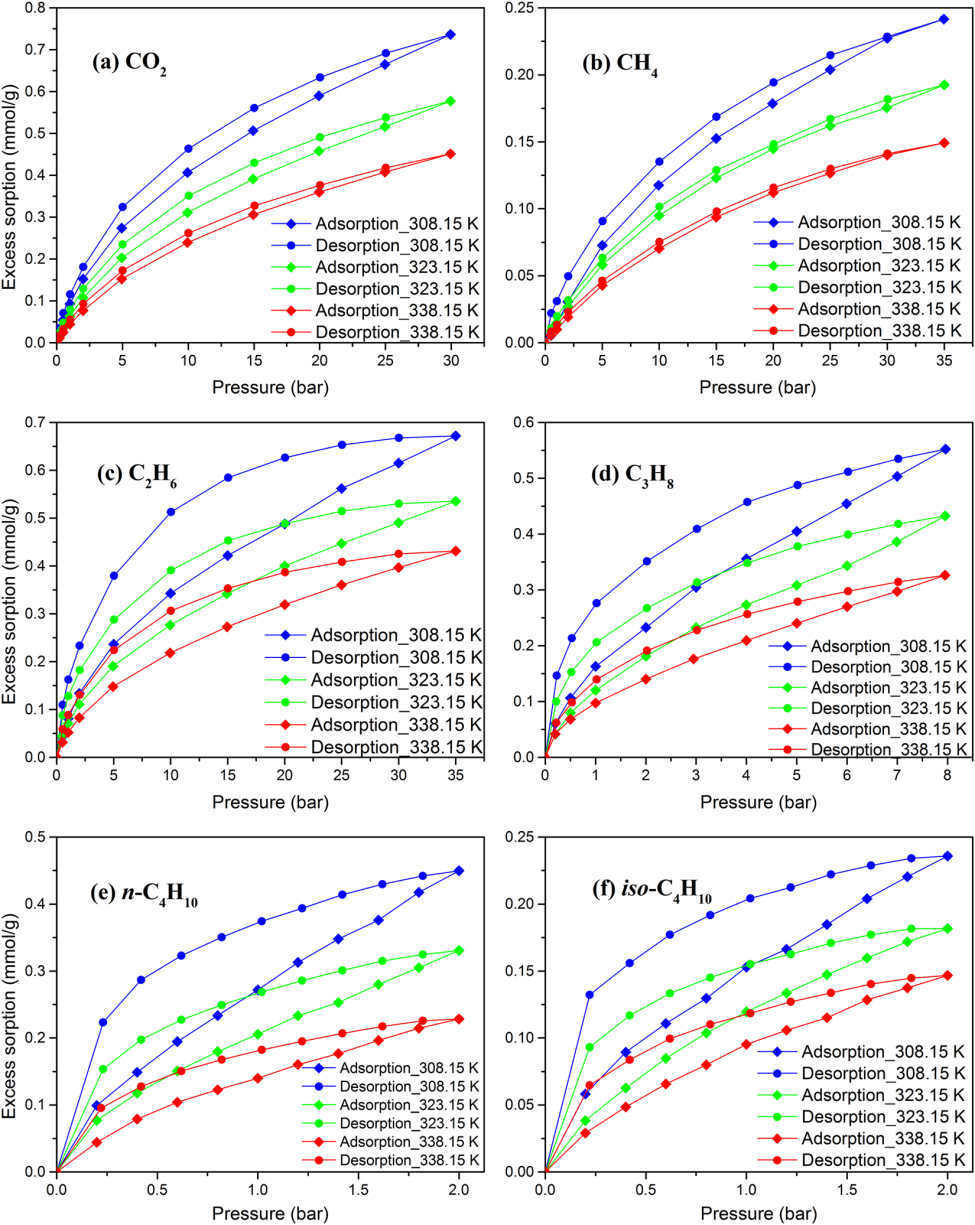
Sorption isotherms of various hydrocarbons and carbon dioxide in Kimmeridge Blackstone powders at three different temperatures.

**Figure 4:**
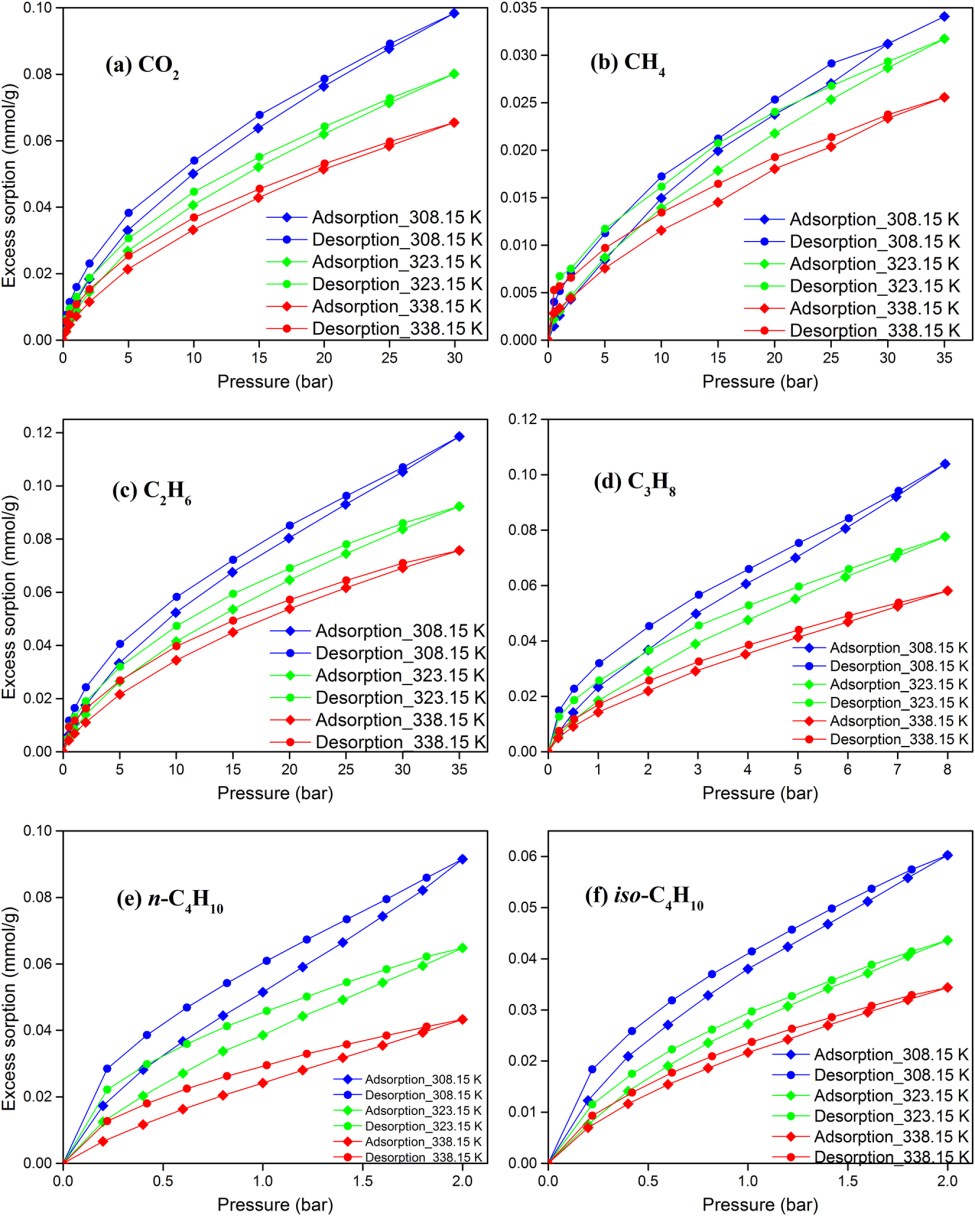
Sorption isotherms of various hydrocarbons and carbon dioxide in Neuquén Shale powders at three different temperatures.

**Figure 5:**
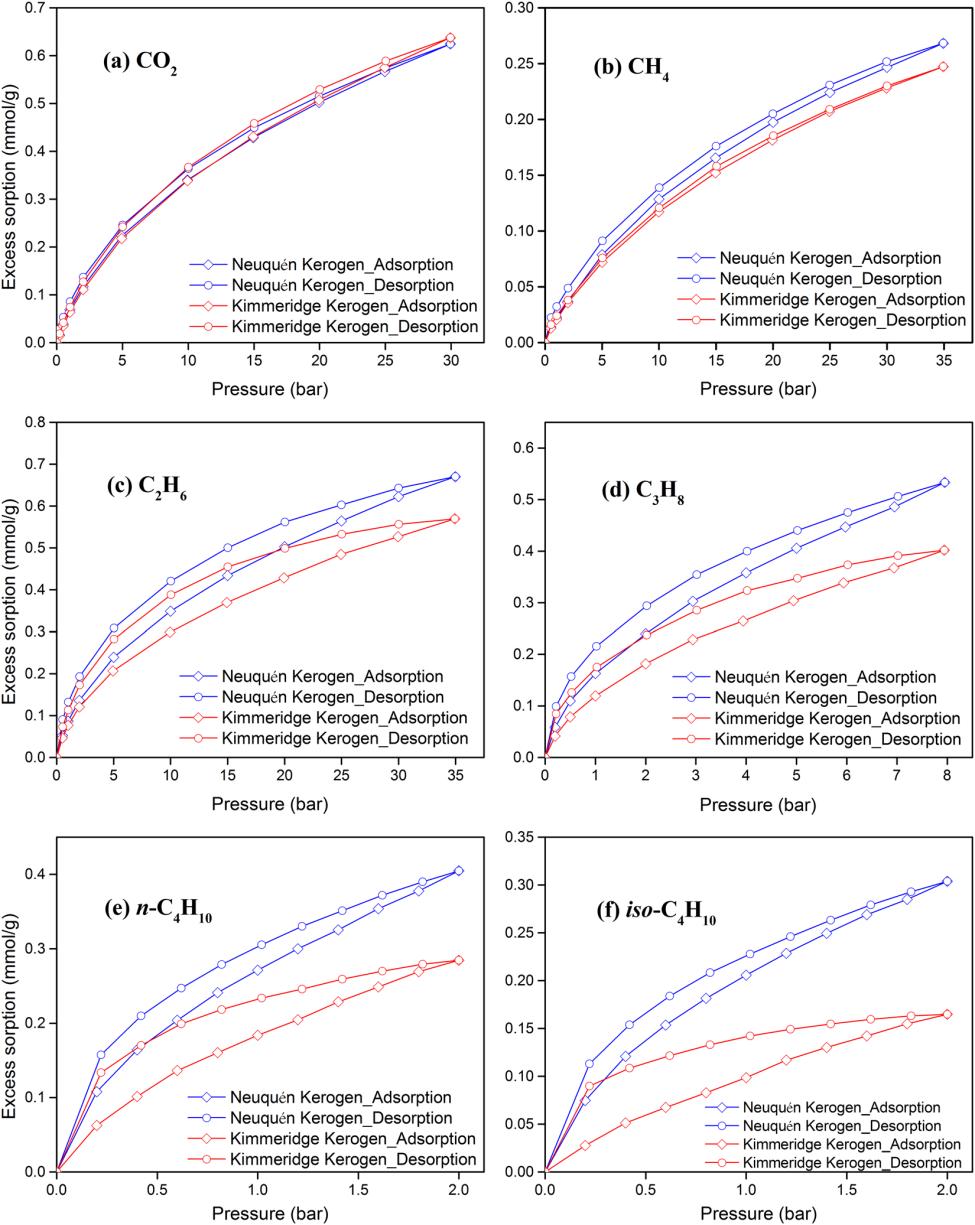
Sorption isotherms of various hydrocarbons and carbon dioxide in the two isolated kerogen powder samples at 338.15 K.

Sorption isotherms of various hydrocarbons and carbon dioxide in two shale powder samples are shown in Figs 3 and 4. These measurements were conducted at three different temperatures, 308.15, 323.15, and 338.15 K. Figures S3 and S4 in the SI show only adsorption data for clarity. Figure 5 shows the sorption isotherms of the two isolated kerogen samples at 338.15 K. The excess sorption presented in Figs 3 to 5 (as well as Figures S3 to S6) refer to the adsorbed species on the inner surfaces of pores and may include dissolved molecules in the kerogen. The expression for the calculation of the excess sorption is presented in the *Methods* section. In the calculation of the excess adsorption presented in this work, the volume of adsorbate and sample swelling are neglected.

## Discussion

The results presented in Figs 3 and 4, as well as Figures S3 and S4 show that adsorption of hydrocarbons and carbon dioxide is higher at low temperatures as expected. Our data show the effect of temperature is more pronounced for propane and butanes than methane. Adsorption of hydrocarbons and carbon dioxide is much higher in Kimmeridge Blackstone which has very high organic matter. For example, at 308.15 K and 35 bar, methane adsorption in Kimmeridge Blackstone is 0.242 mmol/g, which is around 7 times higher than in Neuquén Shale (0.034 mmol/g). As mentioned above, the kerogen content in Kimmeridge Blackstone is 17 times higher than the kerogen content in Neuquén Shale. We cannot accurately identify the surface area and pore volume of kerogen from the shale samples, because once kerogen is isolated from the shale sample by HCl/HF treatment, the pore structure may have been changed. The chemical composition of kerogen also affects the adsorption.

Figures 3 and 4 (Figures S3 and S4) show that *n*-butane has the highest adsorption, followed by *iso*-butane, propane, carbon dioxide, ethane and methane. Butane adsorption can be 10 times more than methane adsorption by mole (37 times by weight) at 2 bar. For the short-chain *n*-alkenes, sorption of the heavier hydrocarbons in shale samples are higher than for the lighter, as expected. Based on molecular simulations, longer carbon chains provide stronger fluid/surface interaction and higher adsorption^4^. *Iso*-butane has a higher vapor pressure than *n*-butane leading to a lesser degree of capillary condensation in the pores. At 293.15 K, the vapor pressures of *n*-butane and *iso*-butane are 2.08 and 3.00 bar, respectively. The shape of hydrocarbon molecules may also affect the fluid/surface interaction. Methane adsorption is around 1/3 of carbon dioxide by mole in both shale samples, which is in line with literature data^6,7,9^. Ethane adsorption is close to carbon dioxide adsorption. In Kimmeridge Blackstone, carbon dioxide adsorption is higher than ethane adsorption. On the other hand, ethane adsorption is somewhat higher than carbon dioxide in Neuquén Shale.

In Figs 3 and 4, ethane, propane, *n*-butane and *iso*-butane show significant hysteresis in adsorption/desorption, especially in Kimmeridge Blackstone which has high organic matter. Even in the sorption isotherms of methane and carbon dioxide, there is a small difference between desorption and adsorption. Capillary condensation of heavier hydrocarbons in the mesoporous shale is thought to contribute to hysteresis^4^. However, the measured hysteresis in Figs 3 and 4 may not be due to capillary condensation only. For the methane sorption, the measured hysteresis cannot be due to capillary condensation. In a typical closed gas sorption hysteresis loop in mesoporous materials, adsorption and desorption isotherms overlap in some pressure range, but there is no overlapping in Figs 3 and 4. Figure S5 shows that the measured hysteresis based on two different runs which provides the standard deviations. The measurements are reproducible in the shale samples. Irreversible structural change of shale sample during the sorption measurements cannot be the reason of hysteresis in this study. Some authors have suggested that change of structure may cause methane and carbon dioxide sorption hysteresis in coal seams^19,29^. Mason *et al*. have reported significant hysteresis in methane adsorption and desorption in the flexible-organic frameworks due to expansion and collapse of the pores with pressure changes^33^. Hysteresis over the entire pressure range in our study may be due to structural change of kerogen. These structural changes are reversible as evidenced in Figure S5. Dissolution of adsorbates into organic matter may also affect the hysteresis.

Total organic carbon contents of Kimmeridge Kerogen and Neuquén Kerogen are close, around 60 wt%. Neuquén Kerogen has much higher surface area (4.5  times) and higher pore volume (4.3 times) than Kimmeridge Kerogen. Carbon dioxide adsorption in Kimmeridge Kerogen and Neuquén Kerogen are similar as shown in Fig. 5. In the hydrocarbons, adsorption in Neuquén Kerogen is higher than in Kimmeridge Kerogen. The difference becomes more pronounced as the number of carbon atoms in the hydrocarbon increases. However, the difference is not proportional to surface area and pore volume. Both the BET surface area and BJH pore volume of Neuquén Kerogen are several times higher than those of Kimmeridge Kerogen. Figure S6 shows that Kimmeridge Kerogen has higher adsorption of various hydrocarbons and carbon dioxide than Kimmeridge Shale (12–68% in various species). Adsorption in Neuquén Kerogen is 8.5 to 10.5 times higher than Neuquén Shale. The increase correlates to some extent with the BET surface area and BJH pore volume increase of 6 times and total organic carbon content increase of 16 times. There is a large difference between the surface areas of isolated kerogen from the two shale samples. The ratio of surface area of Neuquén kerogen to Kimmeridge Kerogen is around 4.5. As Figure S6 indicates the excess sorption does not correlate with surface area. Dissolution in kerogen may result in swelling which can be accounted by change in the volume of the sample in Eq. 4 presented in the *Methods* section. Measurements at higher pressures are warranted for a definitive conclusion.

## Conclusions

In this work we have measured adsorption/desorption of propane, *n*-butane and *iso*-butane in two different shale samples as well as isolated kerogens for the first time. We have also measured adsorption/desorption of methane, ethane and carbon dioxide. Based on our measurements, we draw the following conclusions.

1. Sorption of the hydrocarbons from methane to butanes and carbon dioxide is a strong function of kerogen content and temperature, as expected. The effect of temperature is highest in *n*-butane.
2. Methane adsorption is found to be around 1/3 of the carbon dioxide adsorption by mole, both at pressure of 30 bar. The adsorption of ethane is close to carbon dioxide. Molar adsorption of *n*-butane may be 10 times higher than methane (37 times by mass) at pressure of 2 bar and the same temperature. There is significant difference between adsorption of *n*-butane and *iso*-butane. Adsorption of *n*-butane is higher than adsorption of *iso*-butane to the pressure of 2 bar.
3. Ethane, propane, *n*-butane and *iso*-butane show significant hysteresis in adsorption/desorption over the entire pressure range. Even for methane and carbon dioxide, there are measurable differences. Hysteresis over the whole pressure range may be due to reversible structural changes of organic matter. There is good reproducibility in our measurements.
4. Sorption in the isolated kerogen samples is a function of hydrocarbon molecular size. The sorption of carbon dioxide and ethane is close in the two samples. In ethane the difference in sorption on the two kerogen samples increases. The difference becomes higher in propane and it becomes more pronounced in *iso*-butane.

## Methods

### Sample Preparation

Large chunks of rock were crushed first, and then ground using a ball mill (Planetary Ball Mill PM 200, Retsch). The milled shale powder samples were dry-sieved by a 200 mesh sieve (W.S. Tyler) to collect particles smaller than 75 µm for the subsequent sample characterization and adsorption/desorption measurements.

### Sample Characterization

A Micromeritics ASAP 2420 Accelerated Surface Area and Porosimetry System was used to determine the specific surface area and pore size distributions of the samples. A Thermo Scientific FLASH 2000 CHNS-O Analyzer was used for the elemental analysis, while total organic carbon content in the samples was analyzed by a CM150 Organic, Inorganic, and Total Carbon Analyzer (UIC, Inc.). The mineral composition of the samples (qualitative) was analyzed by a D8 ADVANCE X-ray Diffraction System (Bruker). The XRD quantitative mineralogy analysis and IR analysis were performed by Texray Laboratory Services, and the organic petrography (maceral) and thermal maturity (vitrinite reflectance) were analyzed by the RPS Group. The quantitative XRD analyses of the samples were performed using a Bruker D5000 X-ray diffractometer with Cu Kα radiation source (λ = 1.5405 Å) and silicon drift detector. IR spectroscopy was performed by a Perkin Elmer Spectrum One FT-IR Spectrophotometer with an Attenuated Total Reflectence (ATR) attachment. For the organic petrography, the samples were investigated in white- and UV-light using a Zeiss Axio-Scope A1 at 625x (50x objective, and 1.25 optivar) in immersion oil. White- and UV-light were provided by an X-Cite 120 LED light source. Photographs were captured using a Gryphax camera attached to the Zeiss Axio-Scope A1, and Gryphax image-capture software. All images were then reproduced and enhanced in Microsoft Powerpoint software. A total of 300 counts of both organic and mineral matter were collected for each sample. The RPS methodology for vitrinite reflectance utilizes grey-scale technology that allows for the accurate measurement of small vitrinite particles, like those commonly encountered in shales and mudstones as dispersed organic matter. A minimum of 50 reflectance measurements were taken for each sample. High-resolution black and white photos were taken using a Gryphax digital camera, and grey-scale values measured using Zeiss AxioScope software. The grey-scale values were then translated into %Ro using a mathematical equation of grey-scale values calibrated to a vitrinite reflectance standard. A histogram of all values collected from a sample was then developed to help identify the *in-situ* vitrinite data, and to subsequently calculate a definitive %Ro.

### Kerogen Isolation

Kerogen was isolated from the shale by acid treatment and Soxhlet extraction process^32^. Carbonates were removed by 6 N HCl acid at around 333 K. The remains were filtered and washed with deionized water (DI water) and oven-dried at 353 K. Silicates were removed by a mixture of HCl and HF acids (6 N HCl + 24 wt% HF) at around 333 K, and the remains were filtered and washed with DI water and oven-dried again. Thereafter, Soxhlet extraction with toluene was used to remove extractable organic components. The treated samples were oven-dried under vacuum. All the chemicals used in this study were from Sigma-Aldrich.

### Sorption Measurement

Sorption isotherms of various light hydrocarbons, including methane (99.995%), ethane (99.99%), propane (99.99%), *n*-butane (99.95%) and *iso*-butane (99.95%), and carbon dioxide (99.9999%) in shale samples were investigated using an IsoSORP STATIC (G&V-MP) Automatic Gravimetric High Pressure Sorption Analyzer manufactured by RUBOTHERM. The gases used in this study were from Abdullah Hashim Industrial Gases & Equipment Co. Ltd, Saudi Arabia. Sorption isotherms were determined gravimetrically by weighing the sample using the patented magnetic suspension balance. The gas pressure in the instrument was controlled by a fully automatic pressure controller. Resolution of the magnetic suspension balance is 0.01 mg and the reproducibility is ±0.04 mg (standard deviation).

The sorption measurements comprised four steps. First, the weight (*m* ^*SC*^) and volume (*V* ^*SC*^) of the empty sample container were measured using nitrogen gas (99.9999%) at 308.15 K. Starting from vacuum, the nitrogen pressure was increased stepwise to 30 bar. The decrease of the measured mass of the empty sample container with increasing pressure of the gas phase is due to the buoyancy of the sample container. Based on the linear regression of the measured buoyancy of the empty sample container versus increasing nitrogen gas density, *m* ^*SC*^ and *V* ^*SC*^ were determined. Second, around 1 to 2 g of sample was loaded to the sample container and the sample was vacuum-dried (ultimate vacuum with gas ballast is 0.01 mbar) at 383.15 K until there was no weight change. In the third step, the weight (*m* ^*S*^) and the volume (*V* ^*S*^) of the loaded shale or kerogen sample were determined using helium gas (99.9999%). Starting from vacuum, the helium pressure was increased stepwise to 30 bar at 308.15 K. Based on the linear regression of the measured mass of the loaded sample versus increasing helium gas density, *m* ^*S*^ and *V* ^*S*^ were determined. In the fourth step, the loaded powder sample was evacuated again at 383.15 K until there was no weight change, and then the sorption measurements were conducted. In the adsorption (desorption) measurement, the pressure of the gas was increased (decreased) stepwise at constant temperature. After each sorption measurement cycle, the loaded sample was regenerated by vacuum drying at 383.15 K until there was no weight change.

In the measurements, there are two forces acting on the sample, gravity force (*F*_*A*_) and buoyancy force (*F*_*B*_).

1 $${F}_{A}=({m}^{SC}+{m}^{S}+{m}^{A}).g$$

2 $${F}_{B}=({V}^{SC}+{V}^{S}+{V}^{A}).\rho .g$$

where *m* ^*A*^ and *V* ^*A*^ are weight and volume of adsorbate. ρ is the density of the fluid and g is the gravity acceleration. Balance reading, Δ*m*, and the mass of adsorbate *m* ^*A*^, are given by,

3 $${\rm{\Delta }}m=({F}_{A}-{F}_{B})/g={m}^{SC}+{m}^{S}+{m}^{A}-({V}^{SC}+{V}^{S}+{V}^{A}).\rho $$

4 $${m}^{A}={\rm{\Delta }}m-{m}^{SC}-({V}^{SC}+{V}^{S}+{V}^{A}).\rho $$

Density of the fluid is determined by a sinker, which has known weight and volume, during the measurements. *m* ^*SC*^, *V* ^*SC*^, *m* ^*S*^, and *V* ^*S*^ are measured in the blank measurement and density measurement. The only unknown is *V* ^*A*^, volume of adsorbate. Volume of adsorbate may change during the adsorption and desorption. We report excess sorption based on the assumption that *V* ^*A*^ is zero. We also assume that the sample volume *V* ^*S*^ does not change. In other words, we neglect swelling. Figures 3 to 5, and Figures S3 to S6 were prepared by dividing *m* ^*A*^ by the weight of the sample.

Sorption isotherms were measured based on the equilibrium. For each measuring point, the pressure and temperature were in the range of setting pressure ±0.1 bar and setting temperature ±0.1 °C. If the weight change was smaller than the balance detection limit, 10 μg, in five minutes with pressure and temperature in the range, it was assumed that adsorption/desorption has reached the equilibrium. We repeated a number of measurements, all with good reproducibility. Figure S5 shows two examples.

## Electronic supplementary material

Supplementary Information
