# Supplementary material for: Sorption Hysteresis of Light Hydrocarbons and Carbon Dioxide in Shale and Kerogen
Source: Sci Rep. 2017 Nov 24;7:16209. doi: 10.1038/s41598-017-13123-7 (PMC5701192; doi:10.1038/s41598-017-13123-7)
Supplement: Supplementary file 1 — Supplementary Information [file 41598_2017_13123_MOESM1_ESM.doc]

*Supporting Information*

**Sorption Hysteresis of Light Hydrocarbons and Carbon Dioxide in Shale and Kerogen**

*Huangjing Zhao^1^, Zhiping Lai^2^* and *Abbas Firoozabadi^*1^*

^1^Reservoir Engineering Research Institute, 595 Lytton Avenue Suite B, Palo Alto, CA 94301, USA

^2^Advanced Membranes & Porous Materials Center, King Abdullah University of Science and Technology, Thuwal, 23955-6900, Kingdom of Saudi Arabia

CORRESPONDING AUTHOR FOOTNOTE: Reservoir Engineering Research Institute, Palo Alto, CA 94301, Tel: 650-326-9172, Fax: 650-472-9285, E-mail: [af@rerinst.org](mailto:af@rerinst.org)

**Table of Contents**

**Figure S1.** XRD patterns of shale and kerogen powder samples: (a) Kimmeridge Blackstone; (b) Neuquén Shale; (c) Kimmeridge Kerogen; (d) Neuquén Kerogen.

**Figure S2.** Summary graph of petrographic components of Kimmeridge Blackstone and Neuquén Shale.

**Figure S3.** Adsorption isotherms of various hydrocarbons and carbon dioxide in Kimmeridge Blackstone at three different temperatures.

**Figure S4.** Adsorption isotherms of various hydrocarbons and carbon dioxide in Neuquén Shale at three different temperatures.

**Figure S5.** Reproducibility of adsorption/desorption measurements at 338.15 K: (a) C_2_H_6_ in Neuquén Shale; (b) C_3_H_8_ in Kimmeridge Blackstone. The plots are based on two runs. The standard deviations are small especially in propane where the amount of adsorption in higher than in ethane. The standard deviation in propane is higher at pressures below 1 bar than at higher pressures.

**Figure S6.** Sorption isotherms of various hydrocarbons and carbon dioxide in different shale and kerogen powder samples at 338.15 K.


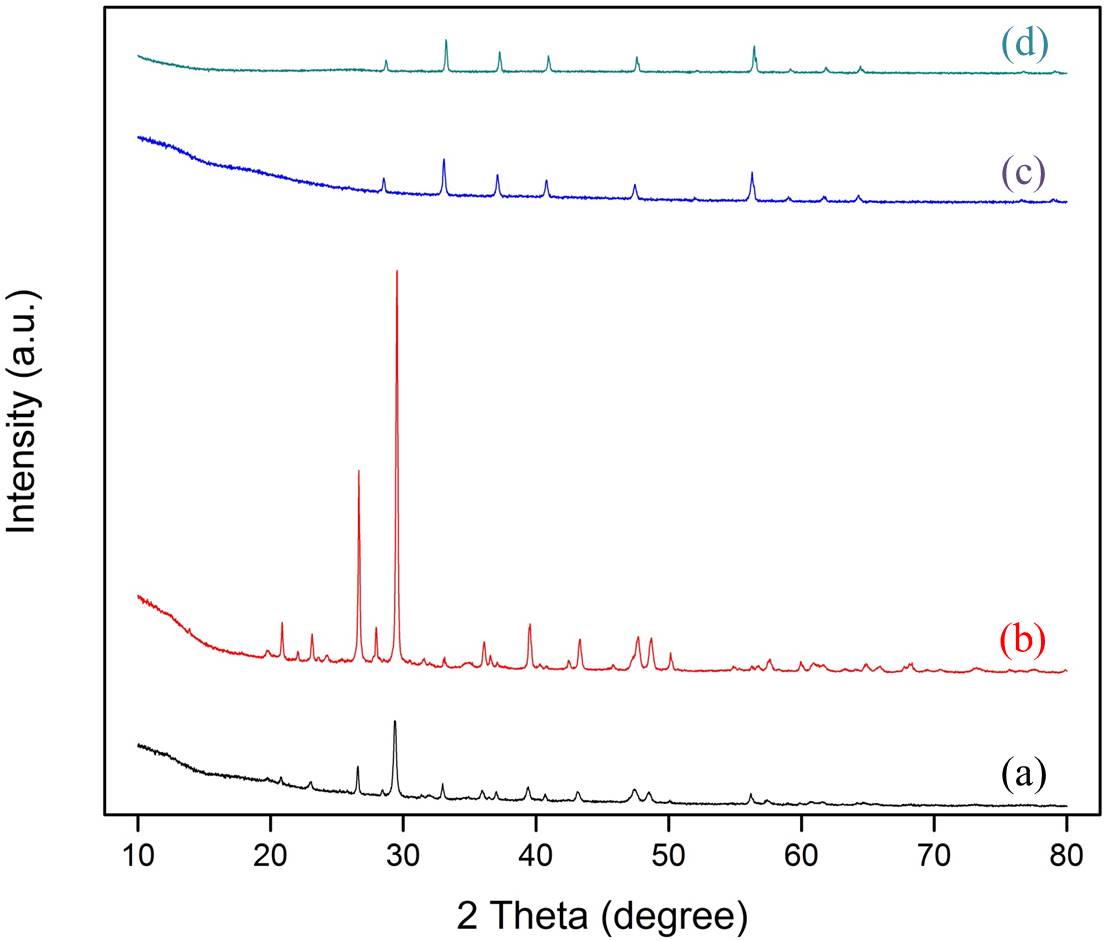


**Figure S1.** XRD patterns of shale and kerogen powder samples: (a) Kimmeridge Blackstone; (b) Neuquén Shale; (c) Kimmeridge Kerogen; (d) Neuquén Kerogen.


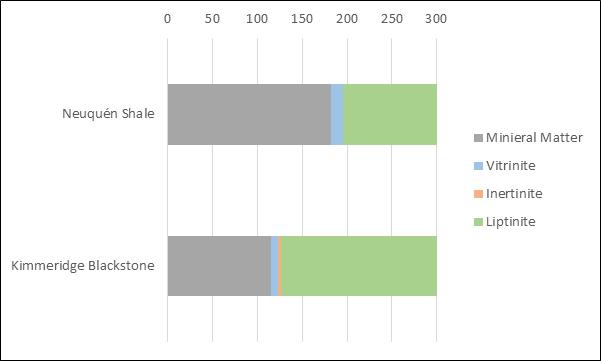


**Figure S2.** Summary graph of petrographic components of Kimmeridge Blackstone and Neuquén Shale.


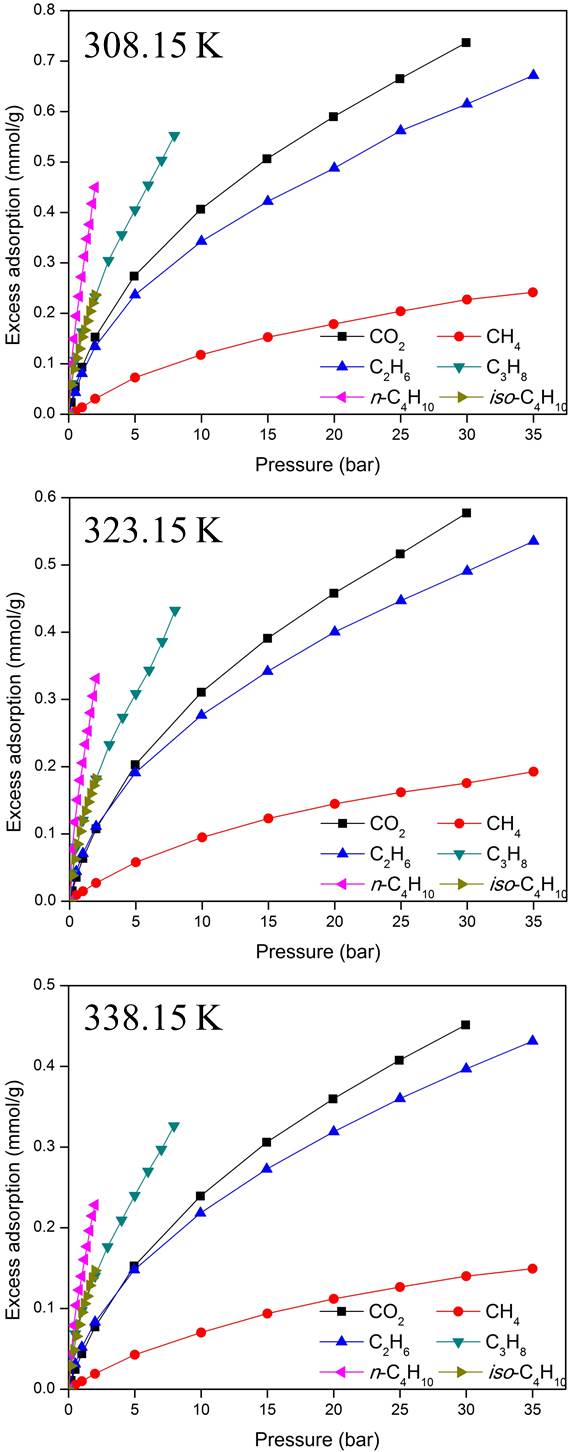


**Figure S3.** Adsorption isotherms of various hydrocarbons and carbon dioxide in Kimmeridge Blackstone at three different temperatures.


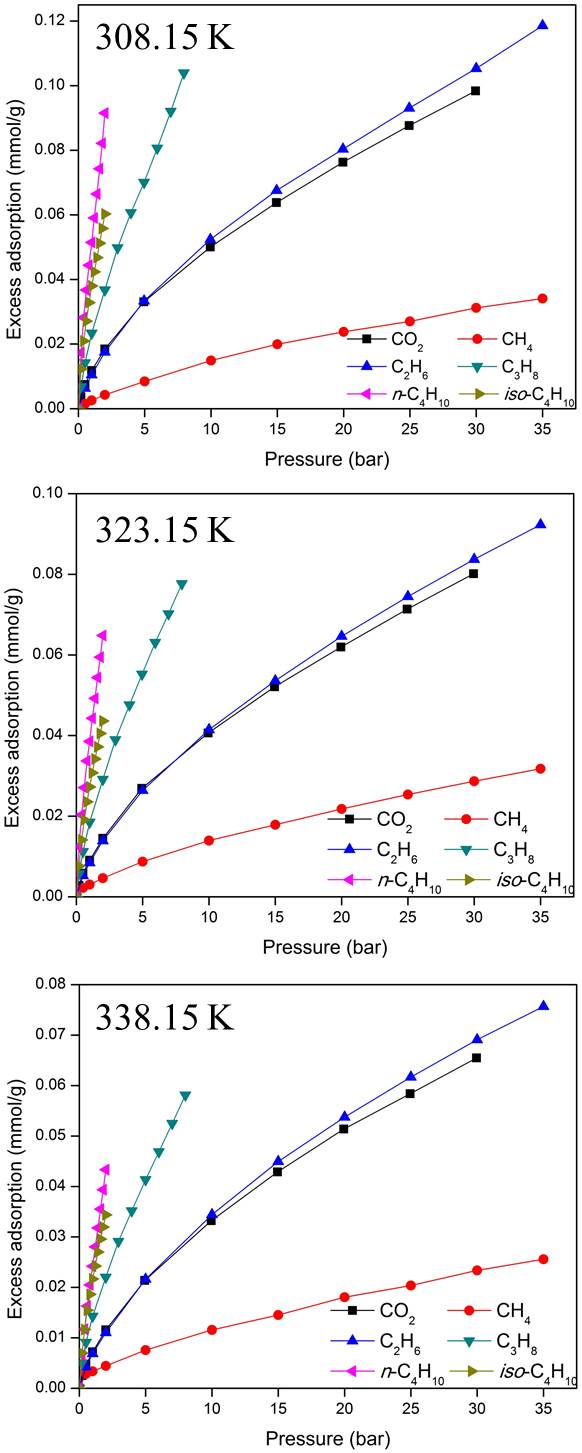


**Figure S4.** Adsorption isotherms of various hydrocarbons and carbon dioxide in Neuquén Shale at three different temperatures.


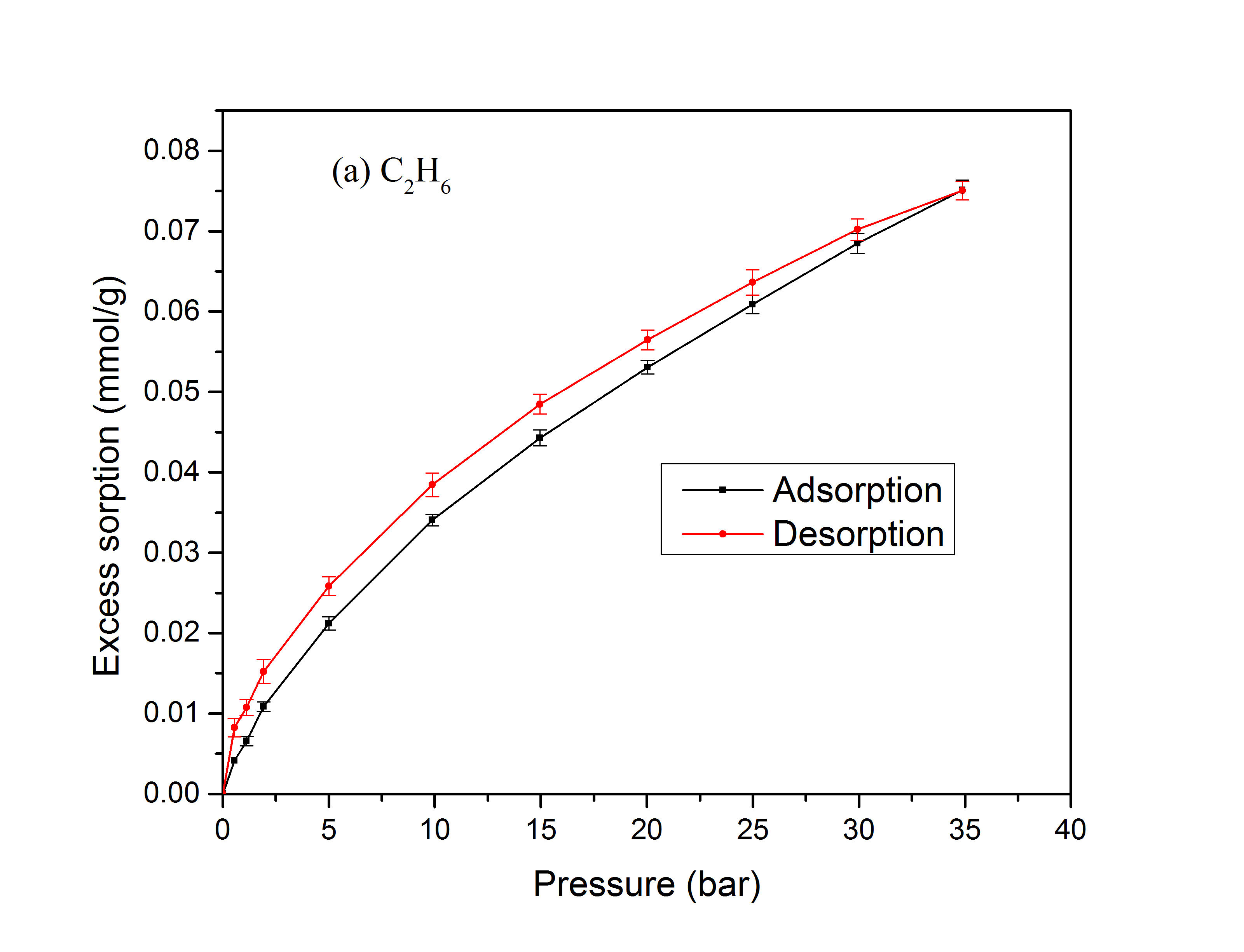


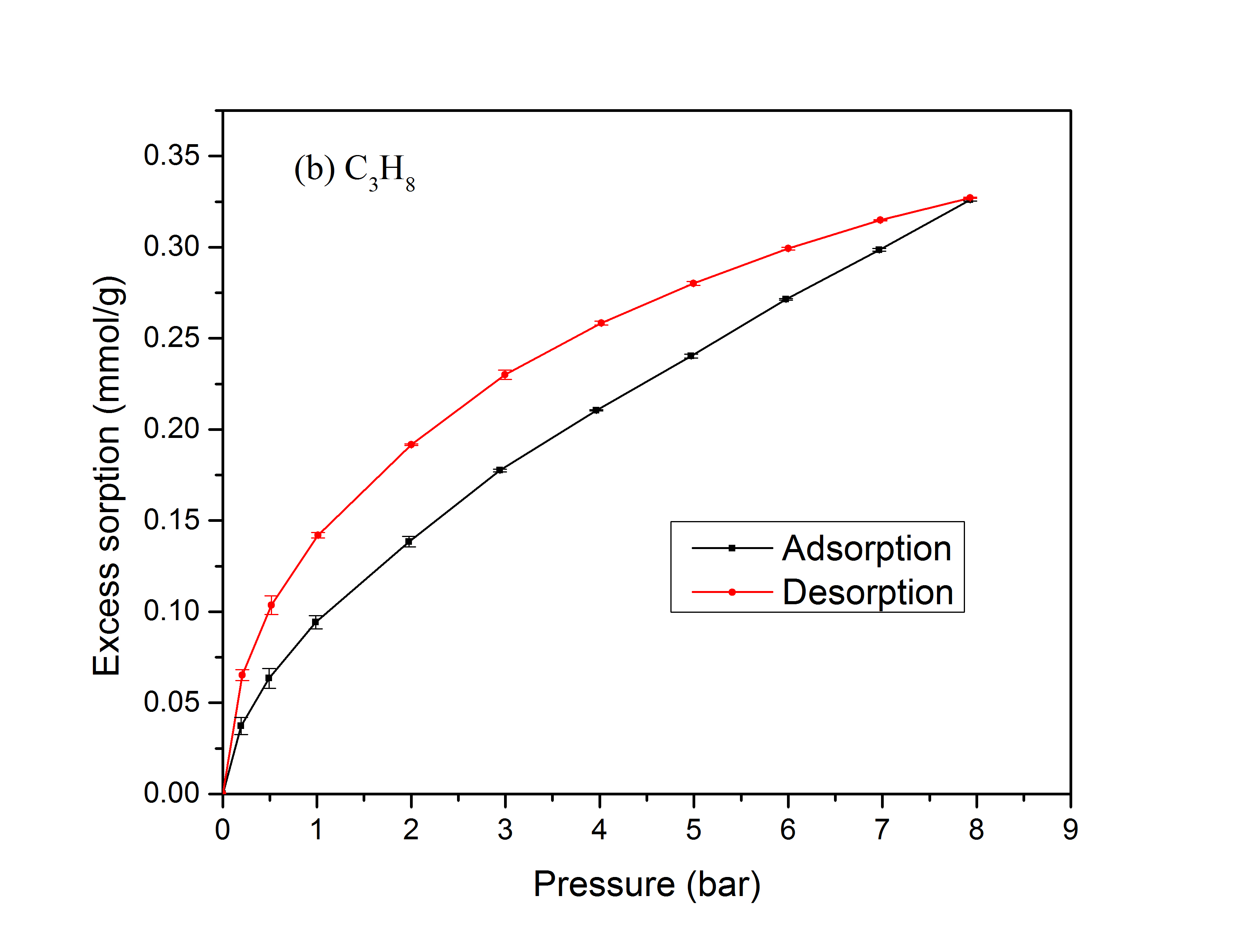


**Figure S5.** Reproducibility of adsorption/desorption measurements at 338.15 K: (a) C_2_H_6_ in Neuquén Shale; (b) C_3_H_8_ in Kimmeridge Blackstone. The plots are based on two runs. The standard deviations are small especially in propane where the amount of adsorption in higher than in ethane. The standard deviation in propane is higher at pressures below 1 bar than at higher pressures.


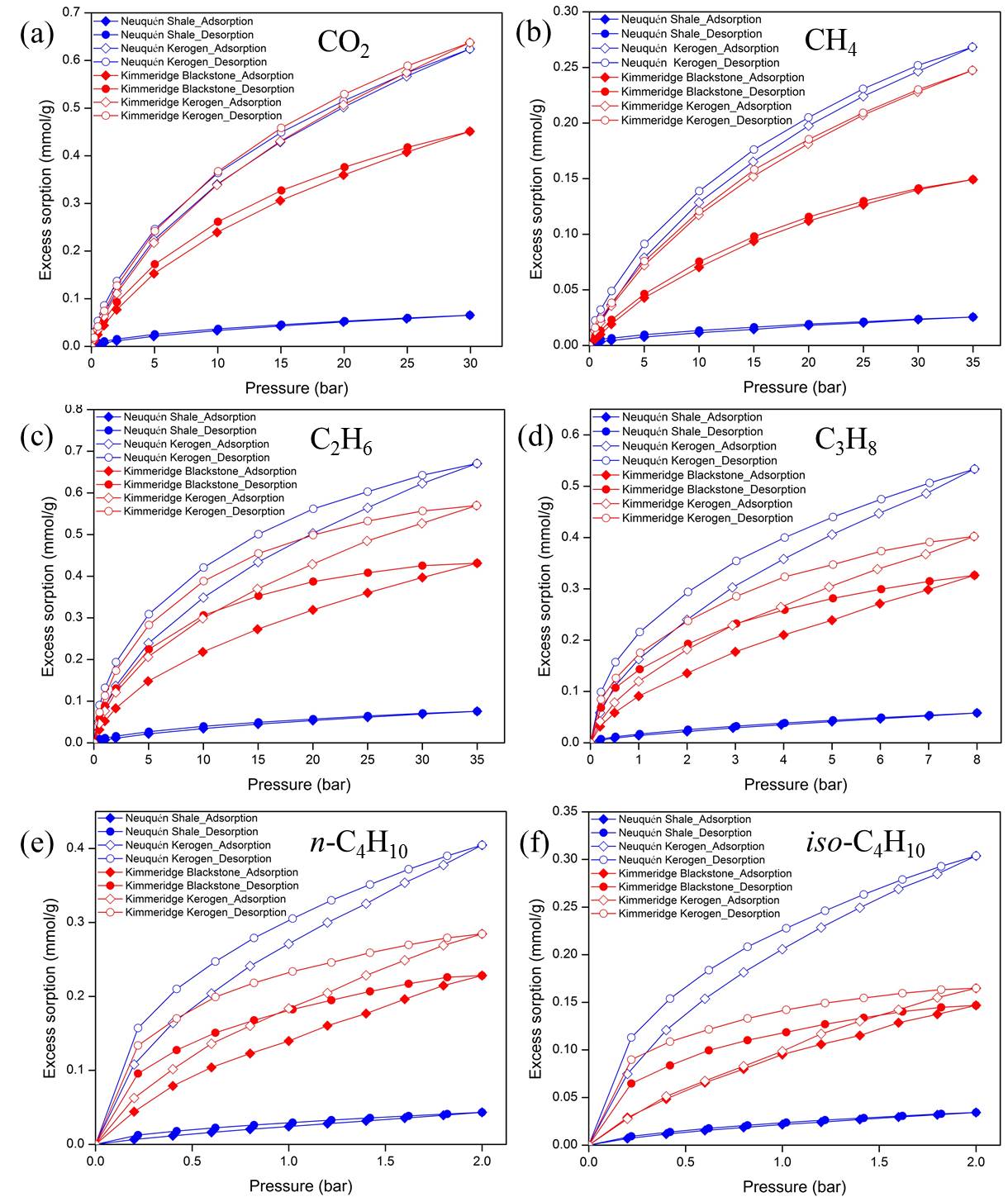


**Figure S6.** Sorption isotherms of various hydrocarbons and carbon dioxide in different shale and kerogen powder samples at 338.15 K.
